# Use of a population-based survey to determine incidence of AIDS-defining opportunistic illnesses among HIV-positive persons receiving medical care in the United States

**DOI:** 10.1186/1742-6405-4-17

**Published:** 2007-09-12

**Authors:** Patrick S Sullivan, Maxine Denniston, AD McNaghten, Susan E Buskin, Stephanie T Broyles, Eve D Mokotoff

**Affiliations:** 1 Centers for Disease Control and Prevention, Division of HIV/AIDS Prevention, 1600 Clifton Road NE, MS E46, Atlanta GA 30333, USA; 2 Public Health – Seattle & King County, 400 Yesler Way, 3rd Floor, SeattleWA 98104, USA; 3 Louisiana Department of Public Health, 2021 Lakeshore Dr. Ste 210, New Orleans LA 70122, USA; 4 Michigan Department of Community Health, 1151 Taylor, Rm 211B Herman Kiefer Health Complex, Detroit MI 48202, USA

## Abstract

**Background:**

Diagnosis of an opportunistic illness (OI) in a person with HIV infection is a sentinel event, indicating opportunities for improving diagnosis of HIV infection and secondary prevention efforts. In the past, rates of OIs in the United States have been calculated in observational cohorts, which may have limited representativeness.

**Methods:**

We used data from a 1998 population-based survey of persons in care for HIV infection to demonstrate the utility of population-based survey data for the calculation of OI rates, with inference to populations in care for HIV infection in three geographic areas: King County Washington, selected health districts in Louisiana, and the state of Michigan.

**Results:**

The overall OI rate was 13.8 per 100 persons with HIV infection in care during 1998 (95% CI, 10.2–17.3). In 1998, an estimated 11.3% of all persons with HIV in care in these areas had at least one OI diagnosis (CI, 8.8–13.9). The most commonly diagnosed OIs were *Pneumocystis jiroveci *pneumonia (PCP) (annual incidence 2.4 per 100 persons, CI 1.0–3.8) and cytomegalovirus retinitis (annual incidence 2.4 per 100 persons, CI 1.0–3.7). OI diagnosis rates were higher in Michigan than in the other two geographic areas, and were different among patients who were white, black and of other races, but were not different by sex or history of injection drug use.

**Conclusion:**

Data from population-based surveys – and, in the coming years, clinical outcomes surveillance systems in the United States – can be used to calculate OI rates with improved generalizability, and such rates should be used in the future as a meaningful indicator of clinical outcomes in persons with HIV infection in care.

## Background

Since the advent of combination antiretroviral therapy (cART), each occurrence of an incident opportunistic illness (OI) in a person with HIV infection is a failure of secondary HIV prevention: new OI diagnoses may represent a failure of early diagnosis of HIV infection, failure to link a diagnosed person to effective medical care, failure to prescribe cART and/or OI prophylaxis when indicated, problems with adherence to cART and/or OI prophylaxis, or drug-resistant HIV infection that is not adequately controlled by prescribed cART. Reliable population-based estimates of OI incidence are thus a high-level measure of multiple goals of prevention and care programs, reflecting both the extent to which persons with HIV are being diagnosed early in the course of disease and entering care in a timely way, and the quality and effectiveness of that care. High overall OI incidence may indicate prevalent problems with late diagnosis of HIV infection or failure to enter care. Some specific OI diagnoses among persons in care may indicate failures of specific OI prophylaxis measures. For example, prophylaxis against *Pneumocystis jiroveci *pneumonia (PCP) and *Mycobacterium avium *complex (MAC) should be provided according to guidelines, and these guidelines may not be strictly followed for various reasons [1]. Understanding the incidence and trends of other OIs may be useful for resource planning, because OIs can be costly to treat.

The present sources of data on OI incidence and prevalence in the United States have significant limitations. Since 1993, when CD4 T-lymphocyte count <200 cells/μL became an AIDS-defining condition in the United States [2] (although not in Europe [3]), US AIDS surveillance programs have seen a decrease in the collection of information on OI diagnoses. Fewer OI diagnoses are reported at the time of AIDS diagnosis because immunologically-defined AIDS usually occurs before an AIDS-OI; over 60% of new AIDS cases in the United States are reported based on CD4 count, with no AIDS-OI [4]. Subsequent OI diagnoses are also undercaptured because, in most US states, surveillance resources are not adequate to conduct followup investigation of OI diagnoses among AIDS cases initially reported as AIDS using the immunological criterion. Although observational cohort studies have served as an important source of information about OI incidence in selected populations, these studies are not representative of all persons in care for HIV infection, and are therefore subject to significant biases [5]. Ideally, population-based data sources should be used to monitor OI incidence over time, to provide an understanding of trends in OIs in the entire population of HIV-infected patients in care.

## Methods

Using data from a 1998 pilot study to demonstrate the use of population-based methods for clinical outcomes surveillance (the Survey of HIV Disease and Care, or SHDC), the incidence of OIs was calculated in three geographic areas in the United States: the state of Michigan, the southern portion of Louisiana (health districts 1,2,3,4, and 9, including New Orleans and Baton Rouge), and King County Washington (including Seattle). The methods of this study have been previously published [6]. In brief, each participating health department constructed a sampling frame of health care providers within the defined geographic area who had ever reported diagnosing or caring for persons with HIV infection to the health department; this list of facilities included both inpatient and outpatient facilities, but excluded sites that only provided HIV testing but not health care (e.g., counseling and testing facilities or laboratories). From this sampling frame, care providers were sampled, using probability proportional to size of the patient population (estimated by the number of persons reported to the health department), after stratification of providers based on size of patient population, urban vs. rural location and whether the provider received support from the Ryan White Program of the Health Resources and Services Administration. To recruit providers to participate, sampled providers were contacted by a variety of methods, including telephone and, in some cases, visits by study staff to the provider's office to explain the study and answer questions the provider may have had.

From each sampled HIV care provider who agreed to participate, the health department requested information about the number and demographic characteristics of patients in care for HIV infection during 1998 in order to create facility-specific patient sampling frames. To be included in the sampling frame, patients were not required to have been previously reported by the provider as an HIV or AIDS case to the health department. Thus, although completeness of reporting of AIDS cases is excellent (>85% in most surveillance areas [7]) any HIV or AIDS cases in care for HIV, but not previously reported, were still eligible for inclusion in SHDC once identified in the provider's office.

Providers used different methods to provide information about patients in care at their practices. For providers with computerized records systems, administrative data were used to provide summary tables of patients in care during the year by race and sex. For providers without such electronic records, manual searches of appointment books or other data sources were conducted. These options for enumerating patients were implemented consistently across study sites, but the availability of electronic records systems largely dictated the choice of methods in any individual practice. Based on this patient information, patients were stratified based on race and sex, and sampled within each provider using systematic sampling within race/sex strata from an ordered list. The sampling interval was varied in the different race/sex strata to ensure adequate representation of women and racial/ethnic minorities.

For each sampled patient, medical records were abstracted for the period January 1, 1998 through December 31, 1998. When the patient had been in care for less than the entire year, the inclusive dates during which the patient was under care at the provider were recorded. Data were collected on clinical diagnoses of AIDS-defining OIs using standard surveillance definitions for definitive and presumptive diagnosis [8]. Abstractors received training in medical records abstraction and HIV including OI definitions and AIDS case definition criteria. Quality assurance procedures (e.g., independent re-abstraction of a small sample of records and/or computerized checks that data were valid [within an expected range]) were implemented in all study areas.

Weights for each patient were calculated by multiplying the sampling weight of the provider and the sampling weight of the patient within the provider as previously described [6]. These weights were used to estimate the numbers of patients in care within the geographic areas, as well as to estimate the numbers of new OI diagnoses during 1998. We estimated annual incidence of OI diagnosis per 100 persons in care for HIV with 95% confidence intervals, and race- and sex-specific OI incidence within each geographic area. For the seven most commonly diagnosed OIs, we also estimated OI-specific incidence in the three geographic areas combined. To allow direct comparison of OI rates from SHDC to OI rates in contemporary observational cohorts, we also estimated incidence density for OIs, by dividing the number of estimated events by the total estimated person-years of observation.

## Results

The SHDC project was considered to be non-research by the Centers for Disease Control and Prevention Institutional Review Board (IRB) and as such did not require IRB review. Of the three participating state and local health departments, the protocol was reviewed and received Institutional Review Board (IRB) approval in two; in one, it was determined to be exempt from IRB review.

Overall, 96% (47/49) of the eligible sampled health care providers agreed to participate in the survey (range by site: 86%-100%). One initially sampled facility was later deemed to be ineligible for participation because the facility had closed. In another case, a sampled provider was later determined to actually represent a professional affiliation of three individual providers. Although specific reasons for not participating were not given by the two providers who refused to participate in King County, study staff felt that the refusals were based on perceived inconvenience and concerns about confidentiality. Also, King County had a local requirement that provider submit a letter of intent to participate, which may have been an impediment to participation.

Information was abstracted from the medical records of 915 patients (range by site: 253–374). Using weighted sums of patients in care, we estimated that our study made statistical inference to 19,761 patients in care for HIV infection in the three geographic areas. Overall, 152 new OI diagnoses were documented in 124 patients in the three areas during 1998. Of these 124 patients, 99 were diagnosed with a single OI, 22 were diagnosed with two different OIs and 3 were diagnosed with 3 different OIs. Based on the 152 observed diagnoses, a total of 2,718 OI diagnoses were estimated in the population during 1998. This represented an annual incidence of OI diagnosis of 13.8 per 100 persons with HIV infection in care during 1998 (95% CI, 10.2–17.3). In 1998, an estimated 11.3% of all persons with HIV in care in 1998 had at least one OI diagnosis (CI, 8.8–13.9). Taking follow time into account, the incidence density of OIs was 35.4 per 100 person-years (p-y; 95% CI, 14.9–55.9).

The most commonly diagnosed OIs were *Pneumocystis jiroveci *pneumonia (PCP) (39 diagnoses observed, 476 diagnoses estimated in the population, annual incidence 2.4, CI 1.0–3.8); cytomegalovirus retinitis (21 diagnoses observed, 464 diagnoses estimated in the population, annual incidence 2.4, CI 1.0–3.7); wasting syndrome (18 diagnoses observed, 243 diagnoses estimated in the population, annual incidence 1.2, CI 0.2–2.3); esophageal candidiasis (11 diagnoses observed, 304 diagnoses estimated in the population, annual incidence 1.5, CI 0.1–3.0); *Mycobacterium avium *complex (10 diagnoses observed, 293 diagnoses estimated in the population, annual incidence 1.5, CI 0.6–2.4); recurrent pneumonia (10 diagnoses observed, 226 diagnoses estimated in the population, annual incidence 1.1, CI 0.2–2.0); and HIV encephalopathy (10 diagnoses observed, 255 diagnoses estimated in the population, annual incidence 1.3, CI 0.1–2.4).

The overall annual incidence of OI diagnosis varied significantly (p = 0.005) across sites: site-specific OI incidence rates were 8.2 (CI, 2.0–14.4) for King County, 8.1 (CI, 4.7–11.5) for southern Louisiana, and 21.9 (CI, 13.0–30.7) for Michigan. Overall, OI rates were different among the three racial/ethnic groups examined in the three areas combined (Table 1). There were no significant differences in OI incidence by sex or history of injection drug use within any of the three geographic areas, and no differences between black- and white-specific OI rates within any area (rates among other races were based on too few events to produce stable estimates for comparison within each area separately).

**Table 1:** Incidence of AIDS-Defining opportunistic illness diagnoses among persons in care for HIV infection in Michigan, Southern Louisiana, and King County Washington – 1998

**Population Group**	**Number of observed persons diagnosed with ≥ 1 OI**	**Estimated total persons diagnosed with ≥ 1 OI (95% CI)**	**Number of observed OI diagnoses**	**Estimated total OI diagnoses (95% CI)**	**Estimated Rate of OI diagnoses per 100 persons in care for HIV (95%CI)**
Overall	124	2236 (1697, 2776)	152	2718 (2158, 3278)	13.8 (10.2, 17.3)
Sex					
Male	97	1760 (1273, 2248)	116	2123 (1365, 2881)	14.5 (9.7, 19.3)
Female	27	476 (210, 742)	36	595 (249, 941)	11.6 (5.3, 17.9)
Race					
White, non-Hispanic	61	898 (571, 1226)	74	1044 (633, 1455)	12.3* (6.8, 17.7)
Black, non-Hispanic	49	1045 (562, 1527)	61	1187 (632, 1742)	11.9* (6.7, 17.1)
Other/unknown race	14	293 (183, 403)	17	487 (195, 779)	38.3* (18.6, 58.0)
History of injecting drug use					
Yes	17	528 (218, 838)	20	687 (201, 1173)	14.9 (6.6, 23.1)
No	107	1708 (1226, 2190)	132	2031 (1485, 2577)	13.4 (9.3, 17.5)

OI: opportunistic illness. * p = .01 for difference by race across all sites

## Discussion

The primary strength of our study is that the patients included were selected using probability sampling methods and are therefore representative of all patients in care for HIV infection in the three participating geographic areas. However, our study also had weaknesses.

The sampling frame of providers was limited to those providers who had reported at least one HIV or AIDS case to the health department; some providers of care may have been left off the sampling frame if they had never reported an HIV case. However, two of the participating states had laboratory reporting of at least some CD4 counts and viral loads at the time of the study and the third had an established, clinically-based HIV reporting system that had been in place and integrated with AIDS surveillance for 10 years. Therefore, HIV care providers, including those who did not provide case reports but who ordered CD4 or viral load tests on patients, would have been known to the health department as providers of HIV care, and were eligible for inclusion in the provider sampling frame.

It is also possible that some patients in care were not appropriately included in the patient sampling frames prepared by the providers, which would have resulted in an incomplete sampling frame at the second stage – but not necessarily in any bias, unless not being included in the sampling frame was related to having had an OI diagnosed during 1998.

In one site, two sampled providers refused participation, which, to the extent that patients in the refusing providers had a different rate of OIs than did patients in care with participating providers, could introduce some bias to our findings. However, because these providers were both in the smallest provider strata, the non-participation of the two providers was unlikely to have introduced a large amount of bias.

Finally, our data reflect only the care and/or diagnosis information included in the medical records of the provider where the patient was sampled. Therefore, for patients who received HIV care from multiple providers, an OI diagnosis that was made outside of the facility where the patient was sampled may not have been recorded. Consequently, our incidence estimates represent minimum estimates of OI incidence. OI ascertainment was retrospective and relatively small numbers of events were observed; therefore, we may have failed to document some OIs which occurred, and the confidence intervals around our incidence estimates were wide in some cases. Moreover, our OI estimates are only representative of patients in care for HIV; however, because many OIs have sufficiently severe clinical presentations, it is likely that persons with these OIs would come to medical attention, even if they had not previously been diagnosed with HIV or were in care for HIV infection. Once they did receive medical care, they would have been included in our sampling frame and could contribute to the OI incidence estimates, even if they had not previously been reported as an HIV or AIDS case to the health department.

This study also had a number of strengths and our estimates of OI incidence differ from most previous estimates in several important ways. First, our estimates are from a probability sample of patients in care for HIV infection. Previous estimates have been reported for patients on therapy before and after the availability of cART [9] and from convenience samples of persons in care for HIV infection [10,11]. Our estimates include all patients in care for HIV infection, regardless of whether they were eligible for cART or whether it was prescribed if indicated. Thus our measure is reflective of OI incidence on a population basis for those in care and is a more appropriate measure of the success of secondary prevention efforts at the population level. How important is this distinction? It is likely that the representativeness of observational cohorts varies by the cohort and, to some extent, by country. In the United States, significant variations in clinical care may exist because of differences in reimbursement sources and in availability of treatment resources in different states [12], and because of varying levels of provider experience with management of HIV infection [13]. In this setting, population-based sampling of patients in care for HIV may be especially important to reduce biases. However, in countries with nationalized health care systems and sophisticated electronic medical information systems, a sampling method such as the one described here might not meaningfully increase the representativeness of data compared with data from electronic medical records.

In some cases, our estimates are comparable to rates estimated from observational cohorts; for example, we estimated that the annual rate of PCP (when expressed as incidence density, to allow direct comparison to estimates from cohort data) was 5.1 per 100 p-y (CI 1.3–9.0). A recent analysis of data from the Adult and Adolescent Spectrum of HIV Disease Project from 1994–2003 estimated PCP incidence density at 6.6 per 100 p-y (CI not reported) [1], and a study of OI incidence in a large HIV clinic reported that the incidence density of PCP in 1997 was 1.9 per 100 p-y (CI, 1.0–3.2) [14]. Our current point estimate for overall OI diagnosis rate in 1998 expressed as incidence density (35.4 per 100 p-y) was somewhat higher than was reported from a large observational cohort in 1997 (14.8 per 100 p-y) [11]. Comparing our rates to previously published rates from cohorts is inherently problematic because OI incidence is largely driven by the CD4 count distribution in the population under observation. However, if our data reflect a different underlying CD4 distribution than previous cohort studies, the importance of using a population-based approach would be validated – i.e., the CD4 distribution in the facility-based cohort would represent a biased set of patients compared to all patients in care for HIV infection.

Our analysis identified differences in OI rates among the three participating geographic areas; higher rates were observed in Michigan than in King County or southern Louisiana. The reasons for these differences are not clear. One possible explanation is that Michigan was the only site to conduct this study in the whole state. A recent analysis of unmet need in Michigan found substantially higher rates of unmet need in out state areas than in the Detroit metropolitan area: 47% vs. 39% [15]. In addition, a summary of the Ryan White CARE Act proposals in the United States during 2006 showed reported unmet need rates are approximately 15% higher in states as a whole than in metropolitan areas [16]. Unmet need is defined as not having CD4 counts or viral loads run or not receiving antiretroviral therapy in a one-year period. Persons with unmet need may be in care more sporadically than those without need. Although our study only included people in care, it did not measure the adequacy of care. If differences between OI rates in metropolitan areas and non-metropolitan areas contributed to the observed differences, this would speak to the importance of having population-based measures of clinical outcomes rather than relying on data collected by cohorts based solely in metropolitan areas in the United States.

Other possible explanations for differences in OI rates by state could relate to chance or systematic differences in the types of facilities sampled (for example, arising from differences in how providers were identified for inclusion on the provider sampling frames), such that facilities where OIs were more likely to be diagnosed and recorded in the records were more likely to be on the sampling frame (and therefore sampled more often) in Michigan. We have previously reported that the proportion of SHDC patients who had advanced disease (CD4 T-lymphocyte count <100 cells/μL or a history of an AIDS-defining OI) was higher in Michigan than in King County or Louisiana [6]. This difference may account for some of the difference in OI rates in Michigan, because the most commonly diagnosed OIs in our study were those that occur in patients with very low CD4 counts.

Starting in 2007, CDC is working with health departments in 19 US states and Puerto Rico to implement an annual, national probability sample of patients in care for HIV infection [5]. This new clinical outcomes surveillance system, called the Medical Monitoring Project (MMP) uses a multi-stage probability sampling approach, including a probability sample of states, a probability sample of providers within selected states, and a probability sample of patients within selected providers [17]. The sampling strategy in MMP is based on the methods used in the study reported here, but aims to improve these methods by using equal probability sampling methods to reduce design effects, by using more sources for constructing the provider sampling frame, and by ascertaining all care received by abstracting medical records at all facilities in which care was received for each enrolled patient. Therefore, future estimates of OI incidence from MMP should be subject to less underestimation than are the results of our pilot study.

Population-based clinical outcomes surveillance systems represent an important source of information about OI diagnoses among HIV-infected persons receiving medical care in the United States, and, by extension, our ability to both diagnose HIV early in the course of infection as well as facilitate entry into adequate care. Key advantages include the ability to draw inference to the population of patients in care for HIV infection, and beginning in 2008, the availability of annual national estimates of OI incidence among those in care [17]. Because the surveillance approach described herein does not include information about HIV-infected people not in care, data from future probability surveys of persons in care for HIV infection will be complemented in the United States by a separate supplemental surveillance system to describe the characteristics of persons with HIV infection who have never entered medical care for HIV [18].

## Abbreviations

AIDS- Acquired Immune Deficiency Syndrome.

cART- Combination antiretroviral therapy.

HIV- Human Immunodeficiency Virus.

OI- Opportunistic illness.

PCP- *Pneumocystis jiroveci *pneumonia.

P-Y- Person-years.

SHDC- Survey of HIV Disease and Care.

## Competing interests

The author(s) declare that they have no competing interests.

## Authors' contributions

PS had primary responsibility for design of the study, for developing the analysis concept, and for writing the manuscript. AM had responsibility for oversight of study operations, and participated in the drafting of the manuscript. MD had responsibility for data management and for the analysis of the data, and participated in drafting the manuscript. SEB contributed to the development of the study methods, was responsible for overseeing the collection of data in the King County site, and participated in drafting of the manuscript. STB contributed to the development of the study methods, was responsible for overseeing the collection of data in the Louisiana site, and participated in drafting of the manuscript. EM contributed to the development of the study methods, was responsible for overseeing the collection of data in the Michigan site, and participated in drafting of the manuscript. All authors read and approved the final manuscript.
